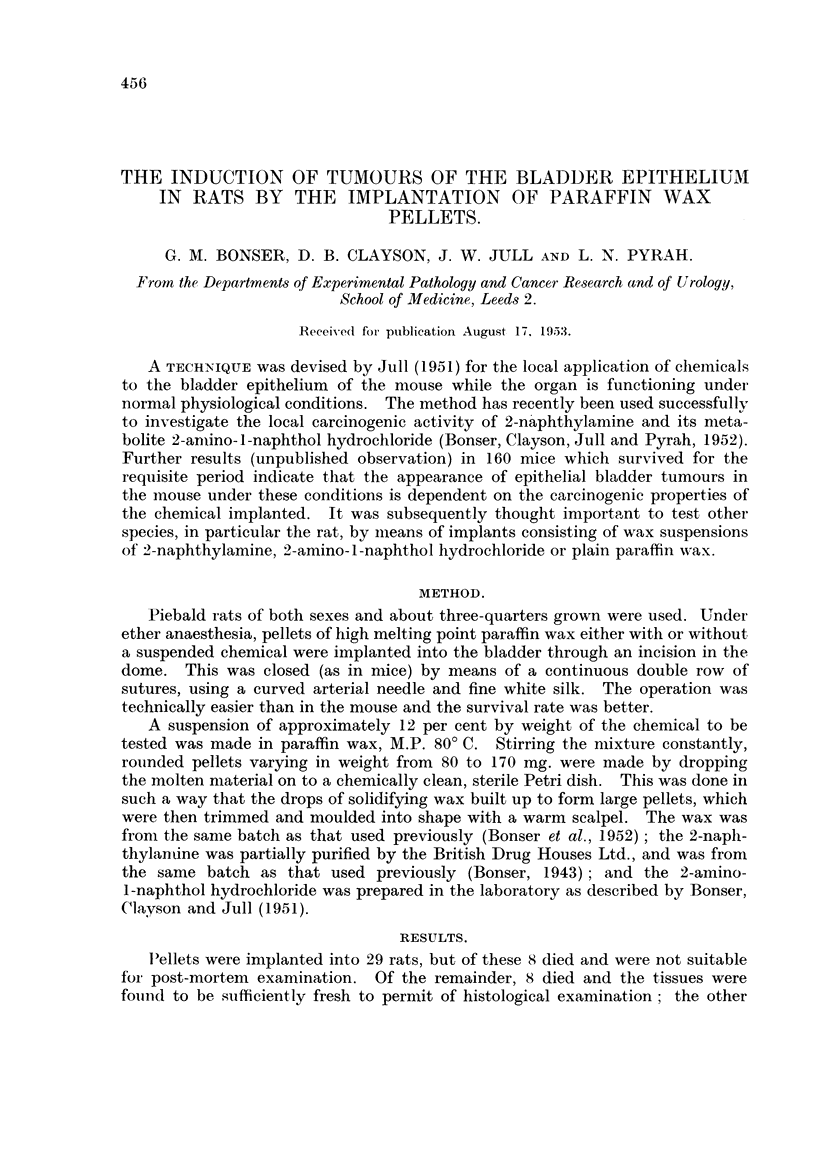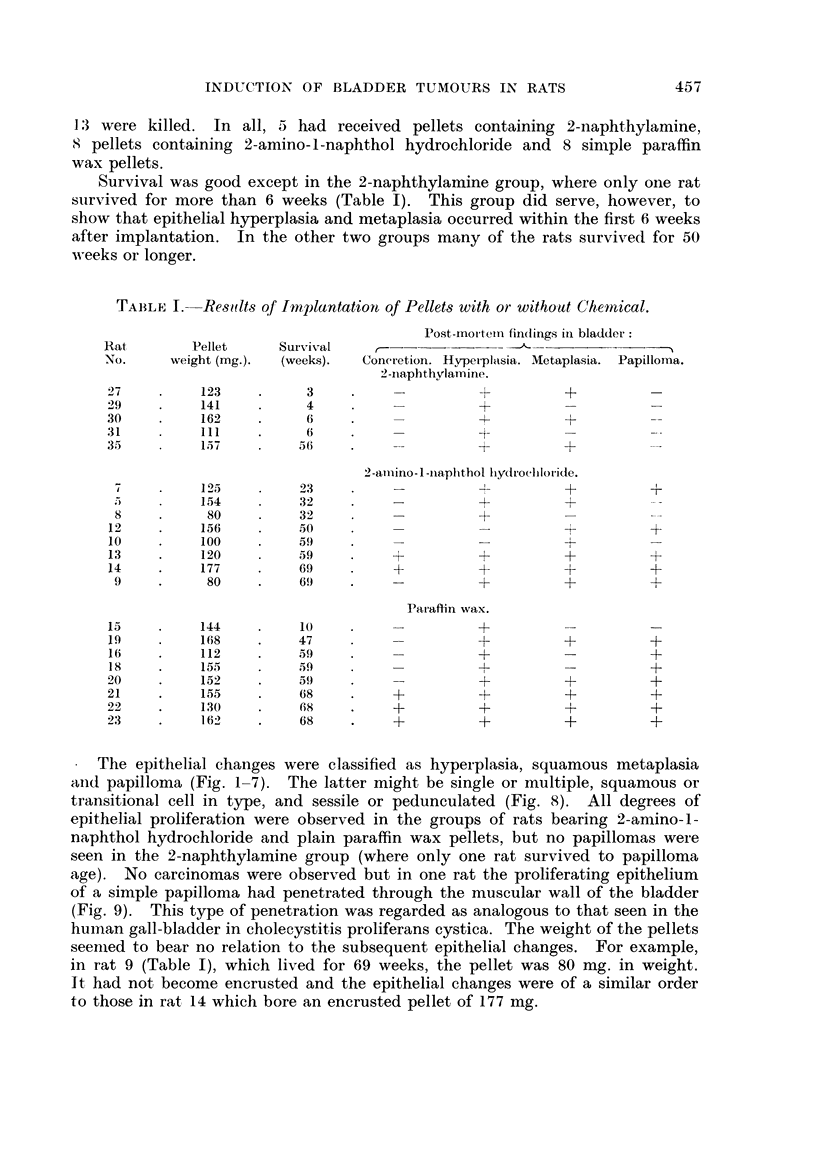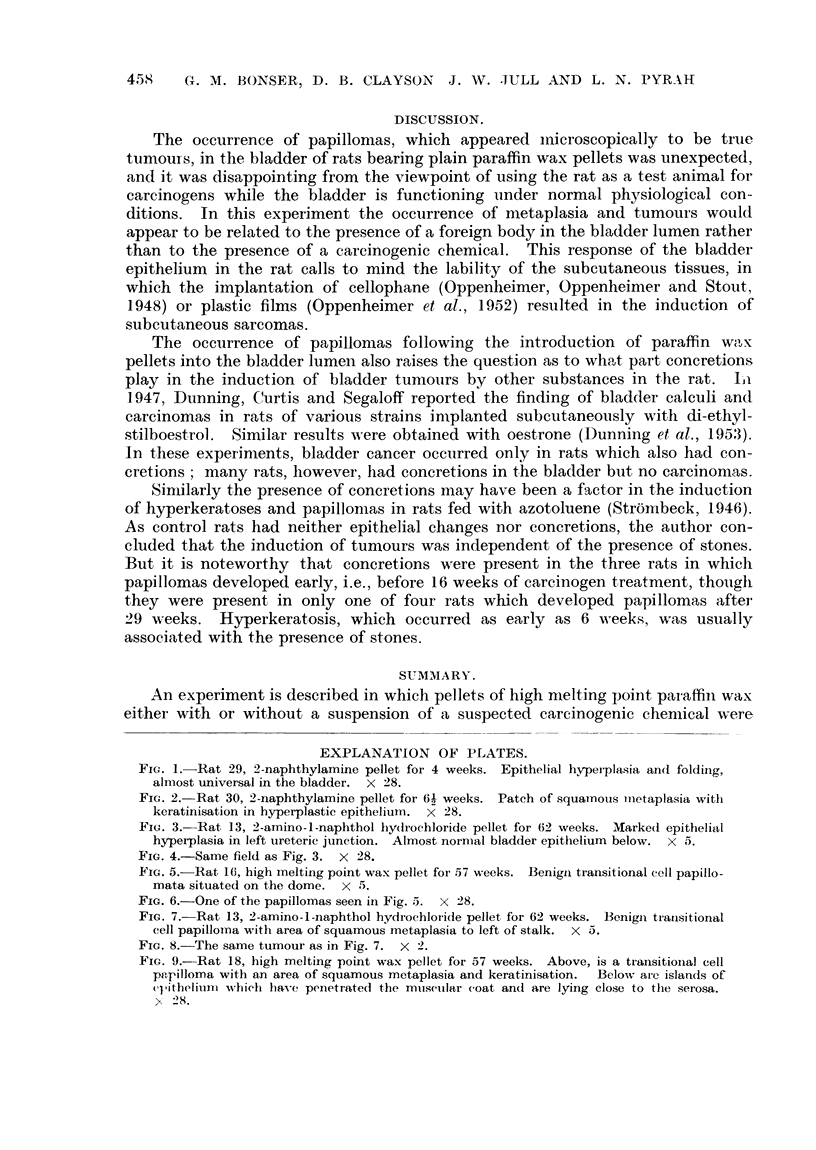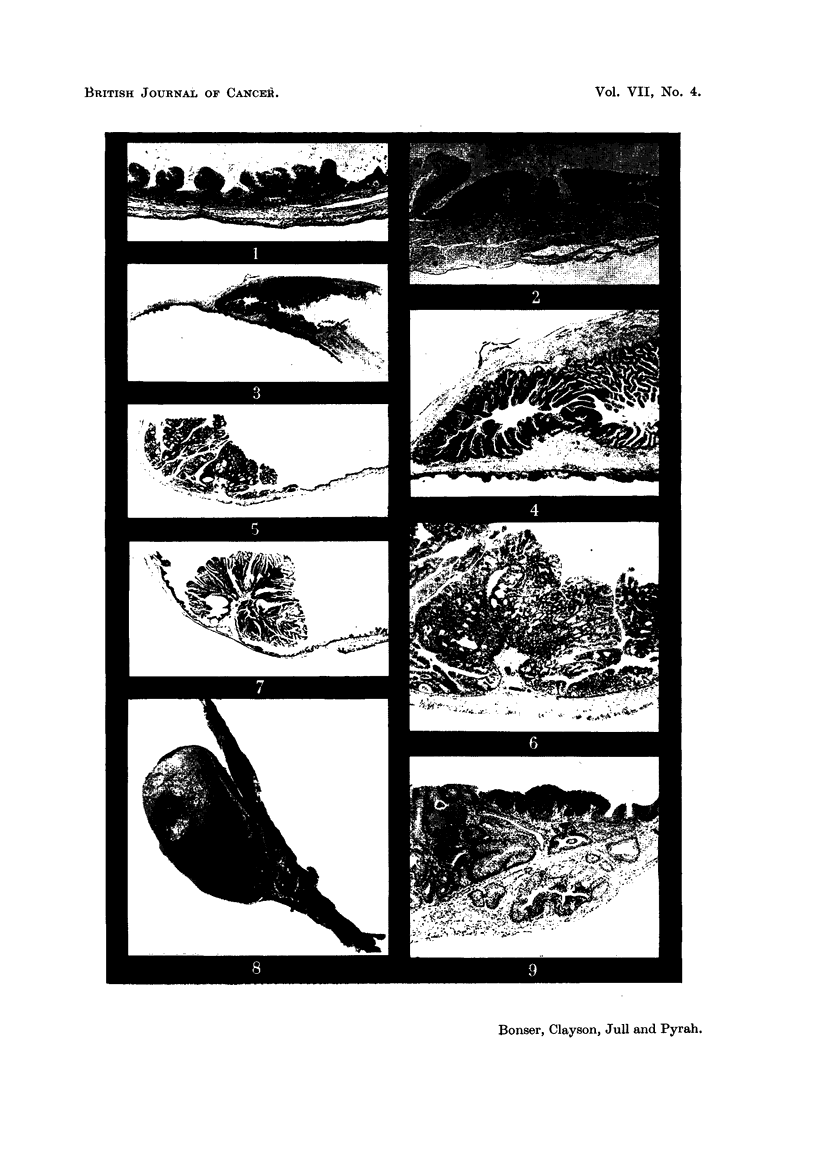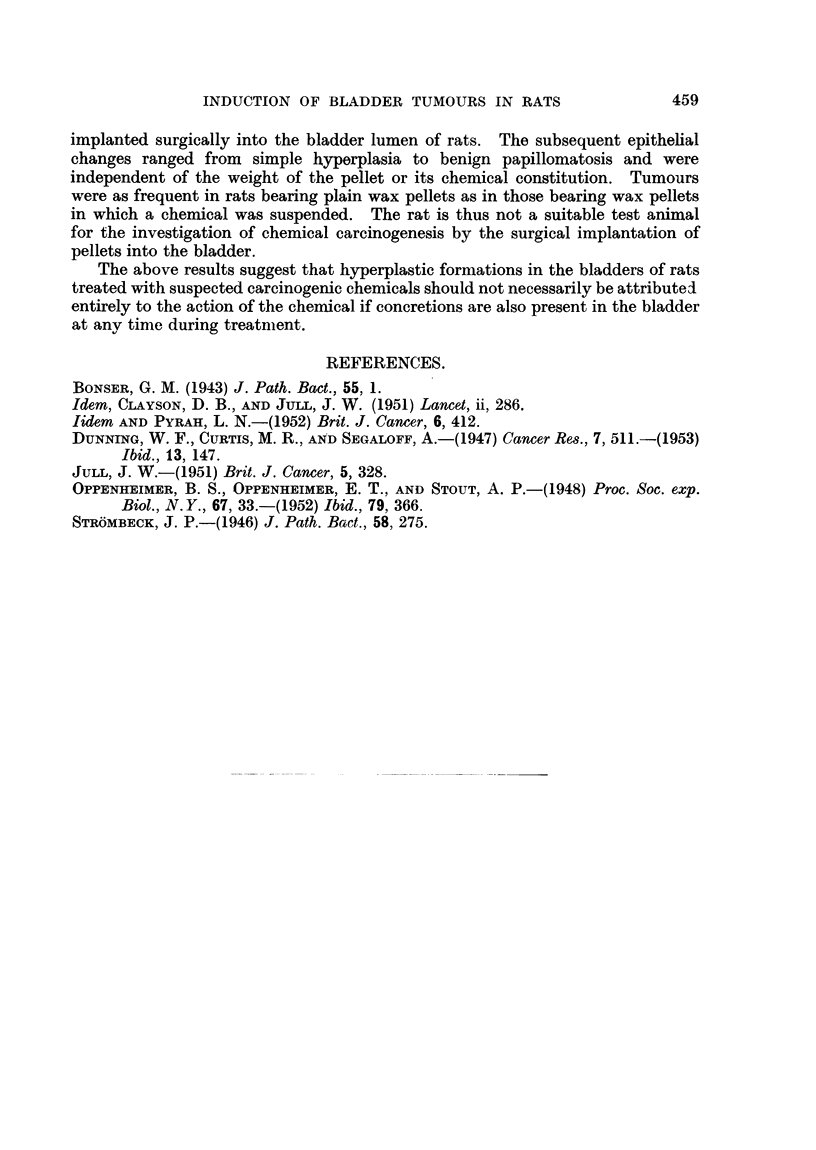# The Induction of Tumours of the Bladder Epithelium in Rats by the Implantation of Paraffin Wax Pellets

**DOI:** 10.1038/bjc.1953.47

**Published:** 1953-12

**Authors:** G. M. Bonser, D. B. Clayson, J. W. Jull, L. N. Pyrah

## Abstract

**Images:**


### THE INDUCTION OF TUMOURS OF THE BLADDER EPITHELIUM IN RATS BY THE IMPLANTATION OF PARAFFIN WAX PELLETS.

G. M. BONSER, D. B. CLAYSON, J. W. JULL AND L. N. PYRAH.

*From the Departments of Experimental Pathology and Cancer Research and of Urology, School of Medicine, Leeds 2.*

Received for publication August 17, 1953.

A TECHNIQUE was devised by Jull (1951) for the local application of chemicals to the bladder epithelium of the mouse while the organ is functioning under normal physiological conditions. The method has recently been used successfully to investigate the local carcinogenic activity of 2-naphthylamine and its metabolite 2-amino-1-naphthol hydrochloride (Bonser, Clayson, Jull and Pyrah, 1952). Further results (unpublished observation) in 160 mice which survived for the requisite period indicate that the appearance of epithelial bladder tumours in the mouse under these conditions is dependent on the carcinogenic properties of the chemical implanted. It was subsequently thought important to test other species, in particular the rat, by means of implants consisting of wax suspensions of 2-naphthylamine, 2-amino-1-naphthol hydrochloride or plain paraffin wax.

### METHOD.

Piebald rats of both sexes and about three-quarters grown were used. Under ether anaesthesia, pellets of high melting point paraffin wax either with or without a suspended chemical were implanted into the bladder through an incision in the dome. This was closed (as in mice) by means of a continuous double row of sutures, using a curved arterial needle and fine white silk. The operation was technically easier than in the mouse and the survival rate was better.

A suspension of approximately 12 per cent by weight of the chemical to be tested was made in paraffin wax, M.P. 80° C. Stirring the mixture constantly, rounded pellets varying in weight from 80 to 170 mg. were made by dropping the molten material on to a chemically clean, sterile Petri dish. This was done in such a way that the drops of solidifying wax built up to form large pellets, which were then trimmed and moulded into shape with a warm scalpel. The wax was from the same batch as that used previously (Bonser *et al.*, 1952); the 2-naphthylamine was partially purified by the British Drug Houses Ltd., and was from the same batch as that used previously (Bonser, 1943); and the 2-amino-1-naphthol hydrochloride was prepared in the laboratory as described by Bonser, Clayson and Jull (1951).

### RESULTS.

Pellets were implanted into 29 rats, but of these 8 died and were not suitable for post-mortem examination. Of the remainder, 8 died and the tissues were found to be sufficiently fresh to permit of histological examination; the other

13 were killed. In all, 5 had received pellets containing 2-naphthylamine, 8 pellets containing 2-amino-1-naphthol hydrochloride and 8 simple paraffin wax pellets.

Survival was good except in the 2-naphthylamine group, where only one rat survived for more than 6 weeks (Table I). This group did serve, however, to show that epithelial hyperplasia and metaplasia occurred within the first 6 weeks after implantation. In the other two groups many of the rats survived for 50 weeks or longer.

TABLE I.—*Results of Implantation of Pellets with or without Chemical.*

| Rat No. | Pellet weight (mg.). | Survival (weeks). | Post-mortem findings in bladder : Concretion. | Hyperplasia. | Metaplasia. | Papilloma. |
|---|---|---|---|---|---|---|
| | | | 2-naphthylamine. | | | |
| 27 | 123 | 3 | − | + | + | − |
| 29 | 141 | 4 | − | + | − | − |
| 30 | 162 | 6 | − | + | + | − |
| 31 | 111 | 6 | − | + | − | − |
| 35 | 157 | 56 | − | + | + | − |
| | | | 2-amino-1-naphthol hydrochloride. | | | |
| 7 | 125 | 23 | − | + | + | + |
| 5 | 154 | 32 | − | + | + | − |
| 8 | 80 | 32 | − | + | − | − |
| 12 | 156 | 50 | − | − | + | + |
| 10 | 100 | 59 | − | − | + | − |
| 13 | 120 | 59 | + | + | + | + |
| 14 | 177 | 69 | + | + | + | + |
| 9 | 80 | 69 | − | + | + | + |
| | | | Paraffin wax. | | | |
| 15 | 144 | 10 | − | + | − | − |
| 19 | 168 | 47 | − | + | + | + |
| 16 | 112 | 59 | − | + | − | + |
| 18 | 155 | 59 | − | + | − | + |
| 20 | 152 | 59 | − | + | + | + |
| 21 | 155 | 68 | + | + | + | + |
| 22 | 130 | 68 | + | + | + | + |
| 23 | 162 | 68 | + | + | + | + |

The epithelial changes were classified as hyperplasia, squamous metaplasia and papilloma (Fig. 1–7). The latter might be single or multiple, squamous or transitional cell in type, and sessile or pedunculated (Fig. 8). All degrees of epithelial proliferation were observed in the groups of rats bearing 2-amino-1-naphthol hydrochloride and plain paraffin wax pellets, but no papillomas were seen in the 2-naphthylamine group (where only one rat survived to papilloma age). No carcinomas were observed but in one rat the proliferating epithelium of a simple papilloma had penetrated through the muscular wall of the bladder (Fig. 9). This type of penetration was regarded as analogous to that seen in the human gall-bladder in cholecystitis proliferans cystica. The weight of the pellets seemed to bear no relation to the subsequent epithelial changes. For example, in rat 9 (Table I), which lived for 69 weeks, the pellet was 80 mg. in weight. It had not become encrusted and the epithelial changes were of a similar order to those in rat 14 which bore an encrusted pellet of 177 mg.

### DISCUSSION.

The occurrence of papillomas, which appeared microscopically to be true tumours, in the bladder of rats bearing plain paraffin wax pellets was unexpected, and it was disappointing from the viewpoint of using the rat as a test animal for carcinogens while the bladder is functioning under normal physiological conditions. In this experiment the occurrence of metaplasia and tumours would appear to be related to the presence of a foreign body in the bladder lumen rather than to the presence of a carcinogenic chemical. This response of the bladder epithelium in the rat calls to mind the lability of the subcutaneous tissues, in which the implantation of cellophane (Oppenheimer, Oppenheimer and Stout, 1948) or plastic films (Oppenheimer *et al.*, 1952) resulted in the induction of subcutaneous sarcomas.

The occurrence of papillomas following the introduction of paraffin wax pellets into the bladder lumen also raises the question as to what part concretions play in the induction of bladder tumours by other substances in the rat. In 1947, Dunning, Curtis and Segaloff reported the finding of bladder calculi and carcinomas in rats of various strains implanted subcutaneously with di-ethylstilboestrol. Similar results were obtained with oestrone (Dunning *et al.*, 1953). In these experiments, bladder cancer occurred only in rats which also had concretions ; many rats, however, had concretions in the bladder but no carcinomas.

Similarly the presence of concretions may have been a factor in the induction of hyperkeratoses and papillomas in rats fed with azotoluene (Strömbeck, 1946). As control rats had neither epithelial changes nor concretions, the author concluded that the induction of tumours was independent of the presence of stones. But it is noteworthy that concretions were present in the three rats in which papillomas developed early, i.e., before 16 weeks of carcinogen treatment, though they were present in only one of four rats which developed papillomas after 29 weeks. Hyperkeratosis, which occurred as early as 6 weeks, was usually associated with the presence of stones.

### SUMMARY.

An experiment is described in which pellets of high melting point paraffin wax either with or without a suspension of a suspected carcinogenic chemical were

---

### EXPLANATION OF PLATES.

FIG. 1.—Rat 29, 2-naphthylamine pellet for 4 weeks. Epithelial hyperplasia and folding, almost universal in the bladder. × 28.

FIG. 2.—Rat 30, 2-naphthylamine pellet for 6½ weeks. Patch of squamous metaplasia with keratinisation in hyperplastic epithelium. × 28.

FIG. 3.—Rat 13, 2-amino-1-naphthol hydrochloride pellet for 62 weeks. Marked epithelial hyperplasia in left ureteric junction. Almost normal bladder epithelium below. × 5.

FIG. 4.—Same field as Fig. 3. × 28.

FIG. 5.—Rat 16, high melting point wax pellet for 57 weeks. Benign transitional cell papillomata situated on the dome. × 5.

FIG. 6.—One of the papillomas seen in Fig. 5. × 28.

FIG. 7.—Rat 13, 2-amino-1-naphthol hydrochloride pellet for 62 weeks. Benign transitional cell papilloma with area of squamous metaplasia to left of stalk. × 5.

FIG. 8.—The same tumour as in Fig. 7. × 2.

FIG. 9.—Rat 18, high melting point wax pellet for 57 weeks. Above, is a transitional cell papilloma with an area of squamous metaplasia and keratinisation. Below are islands of epithelium which have penetrated the muscular coat and are lying close to the serosa. × 28.

Bonser, Clayson, Jull and Pyrah.

implanted surgically into the bladder lumen of rats. The subsequent epithelial changes ranged from simple hyperplasia to benign papillomatosis and were independent of the weight of the pellet or its chemical constitution. Tumours were as frequent in rats bearing plain wax pellets as in those bearing wax pellets in which a chemical was suspended. The rat is thus not a suitable test animal for the investigation of chemical carcinogenesis by the surgical implantation of pellets into the bladder.

The above results suggest that hyperplastic formations in the bladders of rats treated with suspected carcinogenic chemicals should not necessarily be attributed entirely to the action of the chemical if concretions are also present in the bladder at any time during treatment.

### REFERENCES.

Bonser, G. M. (1943) *J. Path. Bact.*, **55**, 1.

*Idem*, Clayson, D. B., and Jull, J. W. (1951) *Lancet*, ii, 286.

*Iidem* and Pyrah, L. N.—(1952) *Brit. J. Cancer*, **6**, 412.

Dunning, W. F., Curtis, M. R., and Segaloff, A.—(1947) *Cancer Res.*, **7**, 511.—(1953) *Ibid.*, **13**, 147.

Jull, J. W.—(1951) *Brit. J. Cancer*, **5**, 328.

Oppenheimer, B. S., Oppenheimer, E. T., and Stout, A. P.—(1948) *Proc. Soc. exp. Biol.*, *N.Y.*, **67**, 33.—(1952) *Ibid.*, **79**, 366.

Strömbeck, J. P.—(1946) *J. Path. Bact.*, **58**, 275.